# Hollow Spherical Heterostructured FeCo‐P Catalysts Derived from MOF‐74 for Efficient Overall Water Splitting

**DOI:** 10.1002/advs.202306919

**Published:** 2023-11-20

**Authors:** Hualin Jiang, Zhe Zhao, Gang Li, Mengxue Wang, Pinghua Chen, Xiaotian Liu, Xinman Tu, Yitian Hu, Zhen Shen, Yirou Wu

**Affiliations:** ^1^ Key Laboratory of Jiangxi Province for Persistent Pollutants Control and Resources Recycle National‐local Joint Engineering Research Center of Heavy Metals Pollutants Control and Resource Utilization Institute of Environmental and Chemical Engineering Nanchang Hangkong University Nanchang 330063 P. R. China; ^2^ Power China Jiangxi Electric Power Construction Co. Ltd. Nanchang 330063 P. R. China; ^3^ Key Laboratory of Jiangxi Province for Persistent Pollutants Control and Resources Recycle Institute of Environmental and Chemical Engineering Nanchang Hangkong University Nanchang 330063 P. R. China

**Keywords:** electricalcatalysts, FeCo‐P, hollow materials, MOF‐74, overall water splitting

## Abstract

The design of catalysts with tunable active sites in heterogeneous interface structures is crucial for addressing challenges in the water‐splitting process. Herein, a hollow spherical heterostructure FeCo‐P is successfully prepared by hydrothermal and phosphorization methods. This hollow structure, along with the heterogeneous interface between Co_2_P and FeP, not only facilitates the exposure of more active sites, but also increases the contact area between the catalyst and the electrolyte, as well as shortens the distance for mass/electron transfer. This enhancement promotes electron transfer to facilitate water decomposition. FeCo‐P exhibits excellent hydrogen evolution (HER) and oxygen evolution (OER) performance when reaching @ 10 mA cm^−2^ in 1 mol L^−1^ KOH, with overpotentials of 131/240 mV for HER/OER. Furthermore, when FeCo‐P is used as both the cathode and anode for overall water splitting (OWS), it only requires low voltages of 1.49, 1.55, and 1.57 V to achieve CDs of 10, 100, and 300 mA cm^−2^, respectively. Density functional theory calculations indicate that constructing a Co_2_P and FeP heterogeneous interface with good lattice matching can facilitate electron redistribution, thereby enhancing the electrocatalytic performance of OWS. This work opens up new possibilities for the rational design of efficient water electrolysis catalysts derived from MOFs.

## Graphical abstract

The synthesis of FeCo‐P with a hollow spherical heterostructure is achieved through hydrothermal and phosphorization methods. The catalyst exhibits high HER/OER activity in alkaline solutions, with an over potential of 131/240 mV. The heterostructure enhances the catalytic reaction rate. FeCo‐P exhibits an ultra‐low OWS potential of 1.49 V at 10 mA cm^−2^.

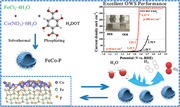

## 1 Introduction

The development of sustainable energy conversion and storage technologies is crucial for mitigating environmental pollution and addressing energy shortages.^[^ ^1^, ^2^, ^3^, ^4^ ^]^ HER and OER are important half‐reactions in the electrochemical water‐splitting process. The OER reaction is a four‐electron transfer process, and its kinetics are slow, which severely hampers the performance of electrochemical hydrogen production.^[^ ^5^ ^]^ Materials based on Pt, Ir, and Ru are currently promising catalysts for HER and OER. However, their high cost and scarcity hinder their widespread application.^[^ ^6^, ^7^, ^8^ ^]^ Therefore, it is of paramount significance to seek efficient, low‐cost metal catalysts as alternatives to precious metal catalysts for water electrolysis.^[^ ^9^, ^10^, ^11^ ^]^

Metal–Organic Frameworks (MOFs) are a class of versatile porous materials constructed from the coordination of metal ions and organic ligands.^[^ ^12^ ^]^ MOFs possess an extremely high specific surface area, unique pore channel structures, and abundantly accessible metal centers, making them ideal candidate materials for electrocatalysts.^[^ ^13^, ^14^ ^]^ However, the application of MOFs in the field of electrochemistry is limited due to their poor chemical/mechanical stability and conductivity. Researches indicate that materials derived from MOFs can enhance their conductivity and stability while preserving the structural diversity and porous characteristics of MOFs.^[^ ^15^, ^16^ ^]^ Additionally, incorporating MOFs into certain composite materials can improve their electrical conductivity.^[^ ^14^ ^]^ For example, Rinawati et al. utilized the synergistic effects between transition metal sites to design a straightforward transformation from bimetallic NiFe‐MOF 74 to NiFe‐LDH. This retained the framework structure of MOF, enabling more effective substrate diffusion. The NiFe‐LDH electrocatalyst derived from MOF‐74 exhibited a low overpotential (*η*) of 299 mV at 10 mA cm^−2^.^[^ ^17^ ^]^ Zhang et al.^[^ ^18^ ^]^ introduced selenium to modulate the morphology of MOF‐74. The coupling of selenide with MOF‐74 resulted in Fe_x_Co_y_NizSe–MOF, which exhibited only a 260 mV overpotential at a current density (CD) of 10 mA cm^−2^ in a 1 mol L^−1^ KOH electrolyte. However, these materials suffer from issues such as poor long‐term stability and insufficient electrocatalytic activity, which limit their large‐scale industrial applications. Transition metal phosphides (TMPs) have emerged as a hot topic in electrocatalysis due to their impressive activity, stability, and conductivity.^[^ ^19^, ^20^ ^]^ Due to their differential adsorption capabilities for various reaction intermediates, most TMPs exhibit excellent HER or OER performance.^[^ ^21^ ^]^ Phosphorus possesses a strong electronegativity, attracting electrons from metal atoms, thus becoming a negatively charged center that attracts positively charged protons to enhance HER activity.^[^ ^22^, ^23^ ^]^ Additionally, the presence of oxygen‐containing functional groups can enhance the hydrophilicity of electrocatalysts, thereby favorably contributing to the improvement of electrocatalytic performance.^[^ ^24^ ^]^ MOF‐74 is an MOF formed by the coordination of divalent transition metals with 2,5‐dihydroxyterephthalic acid (H_4_DOT). Its distinctive features include the wide range of choices and tunability in the composition of divalent metals, as well as varying bond strengths between divalent transition metals and the H_4_DOT ligand. These characteristics make MOF‐74 an ideal precursor for catalysts with extensive tunability.^[^ ^25^, ^26^ ^]^

Besides the chemical components, a material's performance is closely related to its construction. Surface engineering design strategies are commonly used to enhance the electrocatalytic performance of catalysts.^[^ ^27^, ^28^, ^29^ ^]^ Among them, the creation of hollow structures has attracted increasing attention, and the hollow nanostructured materials have emerged as promising candidates with extensive applications in catalytic nitrogen fixation,^[^ ^30^ ^]^ NOx storage,^[^ ^31^ ^]^ photocatalytic degradation,^[^ ^32^ ^]^ energy storage and conversion^[^ ^33^, ^34^, ^35^ ^]^ and more. This is due to their enriched surface area, significantly increased exposed active sites, shortened electron diffusion pathways, low density, rapid mass diffusion rates, and high atomic utilization. Furthermore, the construction of hollow structures can mitigate volume effects, thus enhancing the cyclic stability of the structure.^[^ ^36^, ^37^ ^]^

In this study, the FeCo–MOF‐74 precursor was synthesized using a simple hydrothermal method, followed by a phosphorization process through calcination to produce a hollow spherical FeCo‐P catalyst composed of nanosheets. The resulting hollow structure possesses a larger specific surface area and plenty of accessible active sites.^[^ ^38^, ^39^ ^]^ Furthermore, the hollow structure can enhance the chemical adsorption between the catalyst and active intermediates, further improving catalytic efficiency.^[^ ^40^ ^]^ FeCo‐P exhibits excellent HER/OER and OWS performance. FeCo‐P only requires overpotentials (*η*) of 131/240 mV for HER/OER, with Tafel slopes of 89.90/38.24 mV dec^−1^ @ 10 mA cm^−2^ in 1 mol L^−1^ KOH, respectively. Moreover, it retains its original structural morphology and exhibits excellent stability even after prolonged reactions. When assembling FeCo‐P as the cathode and anode in an electrolysis cell, it achieves current densities of 10, 100, and 300 mA cm^−2^ with only 1.49, 1.55, and 1.57 V in 1 mol L^−1^ KOH, respectively. This catalyst exhibits wonderful industrial potential thanks to its good performance at high current densities. Density Functional Theory (DFT) calculations, XPS (X‐ray Photoelectron Spectroscopy), and electrochemical analyses indicate that the heterostructure formed by FeP and Co_2_P in the hollow spherical FeCo‐P catalyst effectively tunes the local electronic structure. This increases the contact area between the catalyst and the electrolyte while reducing the mass/charge transport length. It establishes a strong and integrated heterogeneous interface, addressing issues related to insufficient electrocatalytic activity, interface contact effects, and poor stability during the catalytic process. The incorporation of externally formed nanosheet hollow spheres promotes the efficient progression of HER and OER reactions, thus facilitating high‐efficiency OWS. This provides insights for the rational design of highly efficient catalysts for OWS.

## 2 Results and Discussion

### 2.1 Characterization of Electrocatalyst

**Scheme 1:**
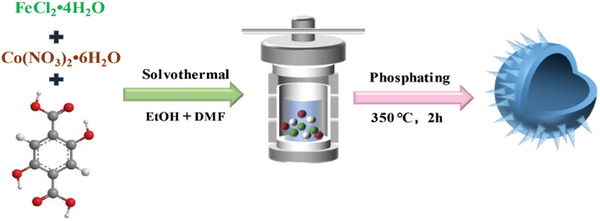
Schematic illustration of synthetic process of FeCo‐P.

**Figure 1:**
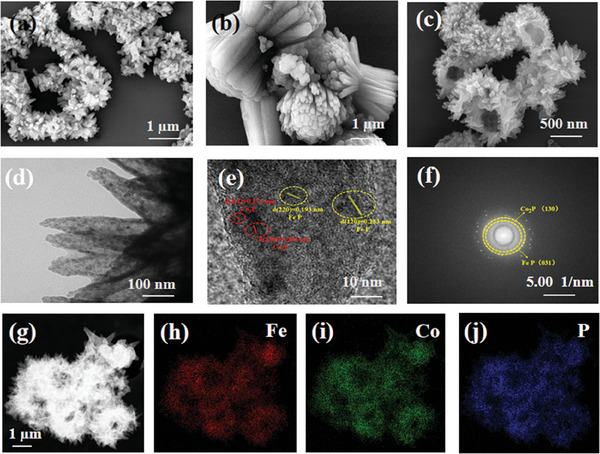
a) SEM images of Fe‐P. b) SEM images of Co‐P. c,d) SEM and TEM images of FeCo‐P. e,f) HRTEM images of FeCo‐P, and g‐j) STEM‐EDS elemental mapping images of the FeCo‐P.

As shown in **Scheme** **1**, we employed a one‐pot hydrothermal method to initially synthesize the precursor FeCo–MOF‐74, followed by phosphorization to obtain FeCo‐P. To further investigate the surface morphology of the prepared samples, scanning electron microscopy (SEM) and transmission electron microscopy (TEM) characterization tests were performed on the electrocatalysts. **Figure** **1**a–c represent the morphology of Fe─P, Co─P, and FeCo─P, respectively. As shown in the figures, Fe─P is a shuttle like structure with a diameter of ≈200 nm, Co─P is a rod‐shaped structure with a diameter of ≈250 nm, and FeCo‐P is a hollow spherical structure composed of nanosheets with a diameter of ≈1 µm. Its hollow structure not only exposes more active sites, but also increases the contact area between the catalyst and electrolyte, which shortens the mass/charge transfer distance. The hollow structure is hence more conducive to the progress of HER and OER reactions. Further analysis of the structural characteristics and chemical composition of FeCo‐P was conducted using TEM. Figure 1d shows a local HR–TEM analysis of the outer nanosheets of the hollow sphere, clearly revealing the morphological structure of the external nanosheets, with small particles formed inside after phosphorization. Figure 1e displays lattice spacings of 0.193 and 0.253 nm corresponding to the (220) and (120) planes of FeP, as well as lattice spacings of 0.271 and 0.205 nm corresponding to the (111) and (130) planes of Co_2_P.^[^ ^18^ ^]^ Moreover, the diffraction rings in Figure 1f clearly reveal the presence of the (130) plane of Co_2_P and the (031) plane of FeP. From the lattice fringe images, it can also be observed that Co_2_P and FeP particles have formed a heterojunction within the nanosheets.^[^ ^19^ ^]^ As shown in Figure 1g–j, EDS analysis of FeCo‐P confirms the uniform distribution of Fe, Co, and P elements within the hollow sphere composed of nanosheets, further substantiating the successful preparation of the FeCo‐P material. The results of the energy dispersion spectrum of FeCo‐P are shown in Figure S9 (Supporting Information), which indicate that the atomic fraction (%) of Co, Fe, and P is 17.27:25.38:57.35 (or 1:1.47:3.32).

The crystal structures of Fe─P, Co─P, and FeCo─P were studied through XRD analysis. As shown in Figure S1 (Supporting Information), the XRD spectrum of Fe‐P exhibits distinct diffraction peaks at 2*θ* = 32.7°, 37.1°, 47.0°, 48.3°, and 56.1°, corresponding to the (011), (111), (220), (211), and (031) planes of FeP (PDF#39‐0809), respectively, consistent with previous reports.^[^ ^41^ ^]^ The XRD spectrum of Co‐P shows distinct diffraction peaks appear at 2*θ* = 32.9°, 37.1°, 48.7°, and 56.2°, which correspond to the (111), (210), (031), and (320) planes of Co_2_P (PDF#32‐0306), respectively, confirming the successful synthesis of Co‐P.^[^ ^42^ ^]^ The XRD spectrum of FeCo‐P exhibits distinct diffraction peaks at 2*θ* = 32.9°, 40.9°, 48.7°, 44.1°, and 52°, corresponding to the (111), (201), (031), (130), and (002) planes of Co_2_P (PDF#32‐0306), respectively. Additionally, at 2*θ* = 32.7°, 35.5°, 37.2°, 47°, 48.3°, and 56.1°, there are evident diffraction peaks, which correspond to the (011), (120), (111), (220), (211), and (031) planes of FeP (PDF#39‐0809), This is consistent with the results from TEM, confirming the successful synthesis of FeCo‐P.^[^ ^43^ ^]^

**Figure 2:**
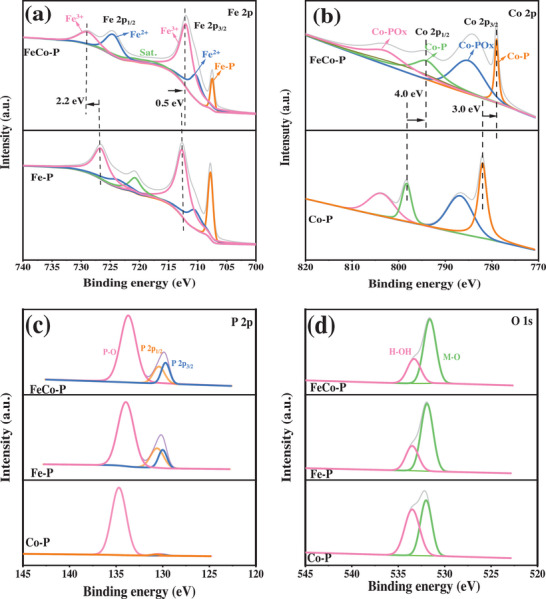
XPS spectrum of Fe─P, Co─P, and FeCo─P: a) Fe 2p spectra, b) Co 2p spectra, c) P 2p spectra, d) O 1s spectra.

The chemical composition and oxidation states of the catalyst were characterized using XPS. As shown in **Figure** **2**a and Figure S2 (Supporting Information), the Fe_2_p_3/2_ spectrum in FeCo‐P can be deconvoluted into three main peaks at 707.4, 710.4, and 712.1 eV, which are attributed to the Fe─P bond, Fe^2+^, and Fe^3+^, respectively.^[^ ^44^ ^]^ The Fe_2_p_1/2_ spectrum in FeCo‐P can be deconvoluted into three main peaks at 720.4, 724.5, and 728.9 eV, respectively, which are attributed to the satellite peak, Fe^2+^, and Fe.^3+[^ ^45^ ^]^ Compared to the monometallic phosphide Fe‐P, the Fe 2p_3/2_ peak in the bimetallic phosphide FeCo‐P shifts by 0.5 eV toward a lower binding energy region, and the Fe_2_p_1/2_ peak shifts by 2.2 eV toward a higher binding energy region. This indicates a significant electron transfer occurring between the heterojunction of bimetallic phosphides Co_2_P and FeP.

From Figure 2b, it is evident that the Co_2_p spectrum exhibits four distinct peaks at 778.9, 783.2, 794.1, and 802.8 eV, corresponding to Co─P, Co─PO_x_, Co─P, and Co─PO_x_, respectively.^[^ ^41^, ^46^ ^]^ Compared to the monometallic phosphide Co─P, the Co_2_p_3/2_ and the Co_2_p_1/2_ peaks in FeCo‐P have shifted by 3.0 and 4.0 eV toward a lower binding energy region, respectively. Taking into account the changes in binding energies for both Fe_2_p and Co_2_p, it can be observed that electrons are transferred from Fe to Co in FeCo‐P, indicating a synergistic effect between Fe and Co bimetallic interaction that further tunes the electronic structure.

As shown in Figure 2c, the P_2_p spectrum can be deconvoluted into three distinct peaks at 129.6, 130.4, and 133.7 eV, respectively, corresponding to P_2_p_3/2_, P_2_p_1/2_, and P─O bonds.^[^ ^44^ ^]^ Figure 2d displays multiple oxygen spectra for O 1 s, with peaks at 531.6 and 533.3 eV, which are attributed to the metal‐oxygen bonds and oxygen from adsorbed water, respectively.^[^ ^47^ ^]^ These XPS experimental results further confirm the successful synthesis of the phosphide. The binding energies of Fe_2_p in FeCo‐P shift toward higher values, while those of Co_2_p shift toward lower values due to the interaction between P and the metal elements. Phosphorous possesses a strong electronegativity, enabling it to attract electrons from the metal atoms and become a negatively charged center, while Fe and Co in FeCo‐P become positively charged centers, promoting the binding of more OH‐ ions. This is favorable for the progression of OER and accelerates HER to produce more H_2_.^[^ ^24^, ^48^ ^]^

### 2.2 Electrocatalytic Properties

#### 2.2.1 HER Performance

**Figure 3:**
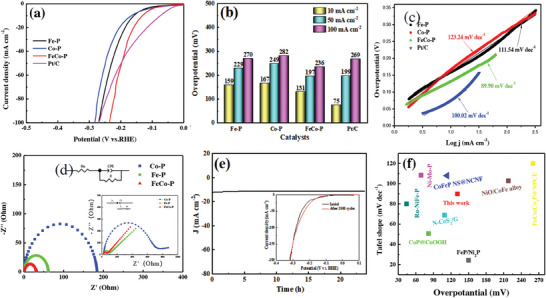
a) HER polarization curves, b) *η* and c) Tafel slope of the as‐prepared electrocatalysts. d) Electrochemical impedance spectroscopy (EIS) of CoP, FeP and FeCo‐P. e) The long‐term stability test of FeCo‐P. f) Comparison of the *η* at a CD of 10 mA cm^−2^ and Tafel slope for FeCo‐P with other state‐of‐art HER catalysts.

The HER performance of the sample was measured in a typical three‐electrode system in a 1 mol L^−1^ KOH solution saturated with N_2_. Based on the LSV (Linear Sweep Voltammetry) in **Figure** **3**a and *η* in Figure 3b, it can be observed that the hollow spherical nanosheets of FeCo‐P exhibit higher HER activity compared to monometallic Fe‐P and Co‐P. FeCo‐P requires an *η* of 131 mV to achieve a CD of 10 mA cm^−2^, which is higher than Pt/C (75 mV) but lower than Fe‐P (159 mV) and Co‐P (167 mV). The above results indicate that bimetallic phosphides are more effective in enhancing electrocatalysts compared to monometallic ones, and the synergistic effect of bimetallic interaction facilitates rapid mass transfer.^[^ ^49^ ^]^ It is worth noting that at current densities greater than 50 mA cm^−2^, the activity of FeCo‐P surpasses that of Pt/C. Furthermore, the kinetic characteristics of the reaction were studied by examining the Tafel slopes. As shown in Figure 3c, the Tafel slopes for FeCo─P, Fe─P, Co─P, and Pt/C are 89.90, 111.54, 123.24, and 100.02 mV dec^−1^, respectively. The Tafel slope for FeCo‐P is lower than those of Pt/C and other samples, indicating its excellent HER kinetic performance.

The electronic transport rate of electrode materials is related to the conductivity of material. Generally, higher conductive electrode materials exhibit faster electronic transport rates. To further understand the material's conductivity, EIS was analyzed. Figure 3d indicates that, through circuit fitting analysis, the impedance plot of FeCo‐P exhibits a characteristic semicircle with the smallest diameter, representing the lowest charge transfer resistance.^[^ ^50^ ^]^ The charge transfer rate of FeCo‐P hollow nanospheres is the fastest, which is attributed to the synergistic effect of bimetallic interaction and the hollow structure that promotes the generation of more active sites while reducing the charge transfer distance,^[^ ^51^ ^]^ thus enhancing the conductivity. The sloping lines in the low‐frequency region reflect Warburg impedance caused by ionic diffusion, with the characteristic that a steep slope favors the migration of ions within the material, while a gradual slope indicates a more pronounced hindrance.^[^ ^52^ ^]^ As observed in the inset of Figure 3d, FeCo‐P exhibits the steepest slope, indicating the highest diffusion migration rate. The impedance results are consistent with the electrocatalytic performance results.

The catalyst's specific surface area and pore size were analyzed using N_2_ adsorption‐desorption isotherms. As shown in Figure S3 and Table S1 (Supporting Information), the specific surface area of Fe─P, Co─P, and FeCo─P are 10.2, 13.6, and 14.3 m^2^ g^−1^, respectively, indicating that they all possess mesoporous structures, and FeCo‐P has the largest specific surface area among them, owing to its unique hollow structure, which increases the catalyst's interface area and is more favorable for both of HER and OER.

In addition, the Turnover Frequency (TOF) value is also an evaluation criterion for catalytic activity.^[^ ^53^ ^]^ TOF values were further calculated at a *η* of 100 mV to assess the intrinsic HER activity of the catalysts. From Table S2 (Supporting Information), it can be observed that the TOF value of FeCo‐P is 0.134 s^−1^, nearly 11 times that of Fe–P (0.012 s^−1^) and 5 times that of Co‐P (0.026 s^−1^) at the same *η*. This further underscores that FeCo‐P exhibits excellent intrinsic HER activity, attributed to the synergistic effect of bimetallic phosphides and the hollow structure of FeCo‐P. In addition to high activity, good stability is also an important parameter for evaluating electrocatalysts. As shown in Figure 3e, the stability of the catalyst was analyzed using chronoamperometry and LSV curves initial and after 2000 cycles. The chronoamperometry curve for FeCo‐P shows no decay in CD at 10 mA cm^−2^ within 24 h, and there is negligible change in the LSV curve initial and after 2000 cycles, indicating that the catalyst exhibits excellent long‐term stability. Furthermore, postreaction SEM analysis revealed that FeCo‐P retained its initial morphological structure, providing additional evidence of its excellent HER stability (see Figure S4, Supporting Information). FeCo‐P's outstanding HER activity surpasses that of some recently reported nonprecious metal HER catalysts, as shown in Figure 3f and Table S3 (Supporting Information).

#### 2.2.2 OER Performance

**Figure 4:**
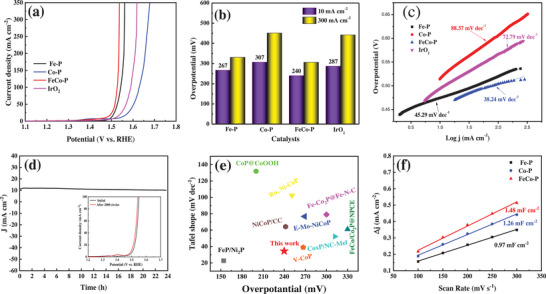
a) OER polarization curves, b) *η* and c) Tafel slope of the as‐prepared electrocatalysts. d) The long‐term stability test of FeCo‐P. e) Comparison of the *η* at a CD of 10 mA cm^−2^ and Tafel slope for FeCo‐P with other state‐of‐art OER catalysts. f) *C*_dl_ of CoP, FeP, and FeCo‐P.

The OER performance of the samples was tested in a N_2_‐saturated 1 mol L^−1^ KOH solution. As shown in **Figure** **4**a,b, the overpotentials for achieving 10 mA cm^−2^ are 240, 267, 307, and 287 mV for FeCo─P, Fe─P, Co─P, and IrO_2_, respectively. FeCo─P demonstrates a significant advantage at high current densities, with an overpotential of only 306 mV at 300 mA cm^−2^. These results indicate that the hollow nanospheres of FeCo─P exhibit higher OER activity compared to monometallic Fe─P and Co─P, and even outperform the precious metal IrO_2_. From Figure 4c, it can be observed that the Tafel slopes for FeCo─P, Fe─P, Co─P, and IrO_2_ are 38.24, 45.29, 88.37 and 72.79 mV dec^−1^, respectively. FeCo‐P hollow nanospheres exhibit the smallest Tafel slope, indicating their excellent OER kinetics.^[^ ^51^ ^]^ For the catalyst's application, long‐term stability is also a crucial factor.^[^ ^54^ ^]^ The OER stability of the samples was evaluated in a N_2_‐saturated 1 mol L^−1^ KOH solution. As shown in Figure 4d, there is negligible change in the LSV curves before and after 2000 cycles. Chronoamperometry curves indicate that the catalyst maintains excellent catalytic activity even after 24 h. Additionally, post‐OER long‐term stability testing SEM analysis (as shown in Figure S5, Supporting Information) reveals no significant changes in morphological structure. FeCo‐P exhibits superior OER activity compared to some recently reported non‐precious metal OER catalysts, as shown in Figure 4e and Table S4 (Supporting Information).

The electrochemical double‐layer capacitance (*C*_dl_) is an important factor in estimating the activity of electrocatalysts. The number of active sites is directly proportional to the electrochemical surface area evaluated by *C*_dl_.^[^ ^55^ ^]^ *C*_dl_ values were calculated by obtaining Cyclic Voltammetry (CV) curves at different scan rates within the non‐Faradaic voltage range (see Figure S6, Supporting Information). From Figure 4f, it can be observed that the *C*_dl_ value for FeCo‐P (1.48 mF cm^−2^) is greater than that of Fe─P (1.26 mF cm^−2^) and Co─P (0.97 mF cm^−2^). This indicates that the bimetallic phosphide FeCo increases the number of active sites more effectively compared to monometallic phosphides, thereby improving HER and OER activities, which consistent with the results of Tafel slopes and impedance.

#### 2.2.3 OWS Performance

**Figure 5:**
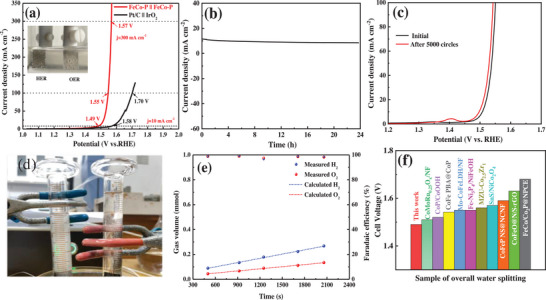
a) Polarization curves of FeCo‐P||FeCo‐P and Pt/C||RuO2 toward overall water splitting. b) Chrono‐potentiometric curves of FeCo‐P||FeCo‐P at ≈10 mA cm^−2^ for 24 h. c) LSV curves of FeCo‐P||FeCo‐P before and after 2000 cycles. d) Gas collection of H_2_ and O_2_. e) FeCo‐P||FeCo‐P Plot of gas production as a function of time during the electrolysis of water. f) Comparison of the required cell voltage @ 10 mA cm^−2^ for FeCo‐P with other state‐of‐the‐art bifunctional electrocatalysts.

To demonstrate the practical application of the FeCo‐P catalyst in an alkaline medium, a bifunctional water splitting device was assembled using FeCo‐P as both the cathode and anode, and tested in a 1 mol L^−1^ KOH solution. Commercially Pt/C was used as the HER electrocatalyst, and IrO_2_ was used as the OER electrocatalyst for comparison. As shown in **Figure** **5**a, the cell voltage for FeCo‐P || FeCo‐P is only 1.49 V@10 mA cm^−2^, significantly outperforming the performance of the precious metal Pt/C || IrO_2_ (1.58 V) for overall water splitting. Furthermore, at 100 and 300 mA cm^−2^, the cell voltages for FeCo‐P || FeCo‐P are 1.55 and 1.57 V, respectively. It can be observed from the data that the FeCo‐P catalyst has a distinct advantage at high current densities, making it highly promising for practical applications. The catalytic performance of FeCo‐P in OWS surpasses that of most reported powder TMP electrocatalysts and some in situ grown phosphide electrocatalysts, as shown in Table S5 (Supporting Information). Stability is another critical criterion for evaluating electrocatalysts. Therefore, the overall water stability of the FeCo‐P electrode was further tested. As shown in Figure 5b,c, the LSV curves after 2000 cycles of CV are essentially identical to the initial LSV curve, indicating excellent durability during the cyclic scanning process. In the long‐term stability test of 24 h at a CD of 10 mA cm^−2^, chronoamperometry curves demonstrate that there is no significant decrease in CD after 24 h, highlighting its excellent stability. Furthermore, SEM characterization of the material after long‐term stability testing in overall water splitting, as shown in Figure S7 (Supporting Information), reveals that the sample still retains its original hollow structure, indicating good structural stability. As depicted in Figure 5d,e, a significant number of bubbles appear on the surfaces of both the cathode and anode during the electrolysis of water. Collection and measurement of these bubbles were performed using a water displacement method. The measured ratio of H_2_ to O_2_ closely approximates the theoretical value of 2:1, and the Faradaic efficiency is close to 100%. A comparison of the OWS performance of FeCo‐P || FeCo‐P with other reported materials is shown in Figure 5f, illustrating the outstanding performance of FeCo‐P || FeCo‐P. These results collectively indicate that the FeCo‐P catalyst exhibits excellent OWS activity and stability, with an voltage of only 1.57 V at a CD of 300 mA cm^−2^, demonstrating its potential for industrial applications.

#### 2.2.4 Density Functional Theory (DFT) Theoretical Calculations

**Figure 6:**
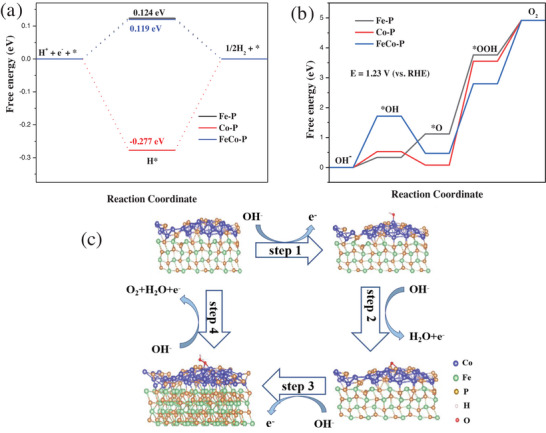
a) Calculated free energy illustration of HER intermediated species. b) Calculated free energy diagram of OER intermediates (U = 1.23 V). c) The OER reaction pathway on FeCo‐P interface.

To better elucidate the mechanisms behind the HER and OER activities of the FeCo‐P catalyst, a series of DFT calculations were conducted. As shown in Figure S8 (Supporting Information), theoretical models for FeP, Co_2_P, and FeCo‐P were constructed. For the alkaline HER process, the activity site with a Δ*G*_H_* (hydrogen adsorption free energy) of 0 attains the best HER activity. In other words, the closer Δ*G*_H_* to 0, the better the catalyst's HER activity.^[^ ^56^, ^57^ ^]^ Calculations yielded Δ*G*_H_* values for FeP, Co_2_P, and FeCo‐P of 0.124, −0.277, and 0.119 eV, respectively (as shown in **Figure** **6**a). This indicates that the Δ*G*_H_* value of heterostructure FeCo‐P is the closest one to 0, facilitating H* adsorption and thus enhancing HER activity. For the OER process in an alkaline electrolyte solution, the computed free energies, as shown in Figure 6b, indicate that the formation of the *OOH intermediate in all the three samples is the slowest step, typically considered as the rate‐determining step, as stabilizing *OOH requires high energy.^[^ ^46^ ^]^ The change in free energy for FeCo‐P is 2.39 eV, which is lower than that for FeP (2.64 eV) and Co_2_P (3.47 eV). The lower energy barrier for the heterostructure compared to the monomers suggests that FeCo‐P exhibits better OER activity. All of the above findings demonstrate that the FeP/Co_2_P heterostructure effectively tunes and optimizes the adsorption abilities of different intermediates in the HER and OER processes, thus enhancing its bifunctional activity. In an alkaline solution, OER involves four proton‐coupled reaction steps, including the adsorption of *OH, *O, and *OOH intermediates. The OER mechanism is illustrated in Figure 6c, where it begins with the adsorption of *OH at the active sites of FeCo‐P, forming HO‐FeCo‐P (Step 1). Subsequently, through deprotonation, oxygen binds into the structure, generating O‐FeCo‐P (Step 2). The exposed oxygen undergoes nucleophilic attack by accepting ‐OH, leading to the formation of *OOH (Step 3). *OOH is further attacked by ‐OH, resulting in the release of O_2_ (Step 4).

## 3 Conclusion

In summary, we prepared a hollow spherical FeCo‐P catalyst composed of nanosheets via a hydrothermal and annealing process and investigated its performance in the HER, OER, and OWS. The hollow structure of FeCo‐P provides abundant active sites, increasing the contact area between the catalyst and the electrolyte, and reducing mass/charge transport distances. Combined with its nanosheet spherical configuration, this structure is highly conducive to the progress of both HER and OER, enabling efficient OWS. The results demonstrate that in 1 mol L^−1^ KOH, FeCo‐P achieves current densities of 10 mA cm^−2^ with overpotentials of only 131 mV for HER and 240 mV for OER, with Tafel slopes of 89.90 and 38.24 mV dec^−1^, respectively. Furthermore, it retains its original structural morphology after long‐term reactions, demonstrating excellent stability. FeCo‐P, when used as both of cathode and anode, achieves low cell voltages of only 1.49 and 1.55 V to reach current densities of 10 and 100 mA cm^−2^, respectively. Additionally, the overall water voltage at a CD of 300 mA cm^−2^ is only 1.57 V, highlighting the industrial potential of this catalyst. This work employs an economical and straightforward approach, providing valuable strategies for the preparation of highly efficient electrocatalysts for energy‐related applications.

## 4 Experimental Section

### Materials

Co(NO_3_)_2_· 6H_2_O and Pt/C (Pt 20%) were obtained from Macklin. FeCl_2_·4H_2_O and anhydrous ethanol were bought from Xilong Chemical Co., Ltd. RuO_2_ and 2,5‐dihydroxyterephthalic acid (H_4_DOT) were sourced from Aladdin. Nafion (5 wt.%) was got from Alfa Aesar. N, N‐dimethylformamide (DMF) was acquired from Tianjin Damao Chemical Reagent Factory. All reagents were used directly. Deionized (DI) water was used throughout the entire experimental process. The nickel foam (NF) substrates were sonicated in 3 mol L^−1^ HCl and DI water for 15 min before use.

### Preparation of FeCo–MOF‐74

H_4_DOT (0.9 mmol), Co(NO_3_)_2_·6H_2_O (0.18 mmol), and FeCl_2_·4H_2_O (0.9 mmol) were added in a mixture of 30 mL DMF and 30 mL ethanol under magnetic stirring until uniform. The resulting mixture was then transferred to a stainless steel high‐pressure autoclave lined with Teflon and maintained at 170 °C for 24 h. After natural cooling, the product was washed with DMF and ethanol and centrifuged, then the final product was obtained through freeze‐drying.

The synthesis of Fe‐MOF‐74 and Co‐MOF‐74 was similar to the method used for FeCo‐MOF‐74, with an equal amount of iron source/cobalt source added in a mixture of 30 mL DMF and 30 mL ethanol. Subsequently, the homogeneous mixture was transferred to a stainless steel high‐pressure autoclave lined with Teflon, and maintained at 170 °C for 24 h. After natural cooling, the product was obtained through washing with DMF and ethanol, centrifugation, and freeze‐drying.

### Preparation of FeCo‐P

A 50 mg of FeCo–MOF‐74 precursor and 1 g of NaH_2_PO_2_•H_2_O were placed separately in ceramic boats. FeCo–MOF‐74 was positioned downstream in the furnace and heated to 350 °C under a nitrogen atmosphere, with a heating rate of 2 °C min^−1^. The mixture was annealed and held at this temperature for 2 h. After cooling to room temperature, FeCo─P was collected. Fe─P and Co─P were synthesized using the same method for comparison.

## Conflict of Interest

The authors declare no conflict of interest.

## Supporting information

Supporting information

## Data Availability

The data that support the findings of this study are available from the corresponding author upon reasonable request.

## References

[1] S. Gupta , M. K. Patel , A. Miotello , N. Patel , Adv. Funct. Mater. 2020, 30, 1906481.

[2] V. Do , P. Prabhu , V. Jose , T. Yoshida , Y. Zhou , H. Miwa , T. Kaneko , T. Uruga , Y. Iwasawa , J. Lee , Adv. Mater. 2023, 35, 2208860. 10.1002/adma.202208860 36598813

[3] F. Yu , Y. Zhou , H. Tan , Y. Li , Z. Kang , Adv. Energy Mater. 2023, 13, 2300119.

[4] X. Zhang , M. Schwarze , R. Schomäcker , R. Krol , F. F. Abdi , Nat. Commun. 2023, 14, 991. 36813780 10.1038/s41467-023-36574-1 PMC9947173

[5] F. Yang , T. Z. Xiong , H. Peng , S. H. Zhou , Q. R. Tan , H. Yang , Y. C. Huang , Chem. Eng. J. 2021, 423, 130279.

[6] X. Li , J. Yin , A. Li , M. Lu , K. Sun , Y. Zhao , D. Gao , F. Cheng , P. Xi , Small 2018, 14, 1801070. 10.1002/smll.201801070 29808557

[7] L. Yan , Z. Xu , X. Liu , S. Mahmood , J. Shen , J. Ning , S. Li , Y. Zhong , Y. Hu , Chem. Eng. J. 2022, 446, 137049.

[8] V. Jose , J. M. V. Nsanzimana , H. Hu , J. Choi , X. Wang , J. Lee , Adv. Energy Mater. 2021, 11, 2100157.

[9] Y. Liu , B. Wang , K. Srinivas , M. Wang , Z. Chen , Z. Su , D. Liu , Y. Li , S. Wang , Y. Chen , Int. J. Hydrogen Energy 2022, 47, 12903.

[10] D. Yang , W. Hou , Y. Lu , W. Zhang , Y. Chen , J. Energy Chem. 2021, 52, 130.

[11] D. Tetzlaff , T. Rensch , L. Messing , P. Banke , S. Grätz , D. Siegmund , L. Borchardt , U. Apfel , Chem. Sci. 2023, 14, 11790. 37920333 10.1039/d3sc04542k PMC10619543

[12] B. Patel , B. Parmar , K. Ravi , R. Patidar , J. C. Chaudhari , D. N. Srivastava , G. R. Bhadu , Appl. Surf. Sci. 2023, 23, 82.

[13] B. Zhang , Y. Zheng , T. Ma , C. D. Yang , Y. Peng , Z. Zhou , M. Zhou , S. Li , Adv. Mater. 2021, 33, 2006042. 10.1002/adma.202006042 PMC11468660 33749910

[14] X. Tang , N. Li , H. Pang , Green Energy Environ. 2022, 7, 636.

[15] B. Wang , Y. Chen , Q. Wu , Y. Lu , X. Zhang , X. Wang , B. Yu , D. Yang , W. Zhang , J. Mater. Sci. Technol. 2021, 74, 11.

[16] Y. Zhang , X. Zheng , X. Guo , J. Zhang , A. Yuan , Y. Du , F. Gao , Appl. Catal. B‐ Environ. 2023, 336, 122891.

[17] M. Rinawati , Y. X. Wang , K. Y. Chen , M. H. Yeh , Chem. Eng. J. 2021, 423, 130204.

[18] X. Zhang , H. Pan , Y. Jia , Y. Zhang , Z. Jiang , C. Li , X. Li , L. Bao , R. Ma , K. Wang , J. Colloid Interface Sci. 2022, 623, 552560. 10.1016/j.jcis.2022.04.181 35598484

[19] X. F. Lu , B. Y. Xia , S. Q. Zang , X. W. Lou , Angew. Chem., Int. Ed. 2020, 132, 4662.

[20] L. M. Cao , J. Zhang , L. W. Ding , Z. Y. Du , C. T. He , J. Energy Chem. 2022, 68, 494.

[21] Y. Y. Liu , Z. H. Xiang , ACS Appl. Mater. Interfaces 2019, 11, 41313. 31613082 10.1021/acsami.9b13540

[22] M. Ramadoss , Y. Chen , X. Chen , Z. Su , M. Karpuraranjith , D. Yang , M. A. Pandit , K. Muralidharan , J. Phys. Chem. C. 2021, 125, 20972.

[23] D. Yang , Z. Su , Y. Chen , K. Srinivas , J. Gao , W. Zhang , Z. Wang , H. Lin , Small 2021, 17, 2006881. 10.1002/smll.202006881 33373091

[24] T. Y. Xu , D. X. Jiao , L. Zhang , H. Y. Zhang , L. R. Zheng , D. J. Singh , J. X. Zhao , W. T. Zheng , X. Q. Cui , Appl. Catal. B‐Eviron. 2022, 316, 121686.

[25] M. J. Qu , Y. M. Jiang , M. Yang , S. Liu , Q. F. Guo , W. Shen , M. Li , R. X. He , Appl. Catal. B‐Eviron. 2020, 263, 118324.

[26] H. Jia , N. Yao , J. Zhu , Y. Liu , Y. Lao , H. Cong , W. Luo , Chin. J. Struct. Chem. 2022, 41, 2208031.

[27] X. Li , C. Li , M. Hou , B. Zhu , W. Chen , C. Sun , Y. Yuan , W. Guan , C. Qin , K. Shao , X. Wang , Z. Su , Nat. Commun. 2023, 14, 5025. 37596263 10.1038/s41467-023-40685-0 PMC10439156

[28] X. Wang , Y. Chen , B. Yu , Z. Wang , H. Wang , B. Sun , W. Li , D. Yang , W. Zhang , Small 2019, 15, 1902613. 10.1002/smll.201902613 31361084

[29] P. Prabhu , V. Jose , J.‐M. Lee , Adv. Funct. Mater. 2020, 30, 1910768.

[30] M. Zhong , Z. Wang , D. Dai , B. Yang , S. Zuo , C. Yao , F. Wu , X. Li , J. Rare Earths. 2022, 40, 586.

[31] W. Liu , H. Cao , Z. Wang , C. Cui , L. Gan , W. Liu , L. Wang , J. Rare Earths. 2022, 40, 626.

[32] W. Luo , Q. Chen , L. Ji , X. Peng , G. Huang , J. Rare Earths. 2022, 40, 605.

[33] X. Wang , B. Wang , Y. Chen , M. Wang , Q. Wu , K. Srinivas , B. Yu , X. Zhang , F. Ma , W. Zhang , J. Mater. Sci. Technol. 2022, 105, 266.

[34] D. Yang , Z. Su , Y. Chen , Y. Lu , B. Yu , K. Srinivas , B. Wang , W. Zhang , J. Mater. Chem. A 2020, 8, 22222.

[35] X. Wang , H. Huang , J. Qian , Y. Li , K. Shen , Appl. Catal. B‐Environ. 2023, 325, 122295.

[36] J. Wang , Z. Wang , D. Mao , D. Wang , Sci. China Chem. 2022, 65, 7.

[37] W. Han , Y. Wei , J. Wan , N. Nakagawa , D. Wang , Inorg. Chem. 2022, 61, 5397. 35312311 10.1021/acs.inorgchem.2c00253

[38] S. Y. Gao , X. F. Lu , W. L. Sim , S. L. Zhang , X. Wen , Angew. Chem., Int. Ed. 2021, 133, 23067.

[39] L. F. Shen , L. Yu , H. B. Wu , X. Y. Yu , X. G. Zhang , Nat. Commun. 2015, 6, 6694. 25798849 10.1038/ncomms7694

[40] Y. Y. Zhai , X. R. Ren , J. Q. Yan , S. Z. Liu , Small Struct. 2021, 2, 2000096.

[41] F. L. Wang , X. D. Yang , B. X. Dong , X. Yu , H. G. Xue , L. G. Feng , Electrochem. Commun. 2018, 92, 33.

[42] H. Yu , D. H. Chua , ACS Appl. Mater. Interfaces 2018, 10, 14777. 29633825 10.1021/acsami.8b02755

[43] J. W. Zhu , J. Q. Chi , T. Cui , L. L. Guo , S. Q. Wu , B. Li , Appl. Catal. B‐Eviron. 2023, 328, 122487.

[44] D. Li , Z. Y. Li , R. Zou , G. Shi , Y. M. Huang , W. Yang , W. Yang , C. F. Liu , X. W. Peng , Appl. Catal. B‐Eviron. 2022, 307, 121170.

[45] M. L. Gao , P. P. Gao , T. Lei , C. O. Yang , X. B. Wu , A. R. Wu , Y. Du , Colloids Surf. A Physicochem. Eng. Asp. 2022, 651, 129673.

[46] Y. Y. Song , J. L. Cheng , J. Liu , Q. Ye , X. Gao , J. J. Lu , Y. L. Cheng , Appl. Catal. B‐Eviron. 2021, 298, 120488.

[47] Y. X. Li , J. Yin , L. An , M. Lu , K. Sun , Y. Q. Zhao , D. Q. Gao , F. Y. Cheng , P. X. Xi , Small 2018, 14, 1801070. 10.1002/smll.201801070 29808557

[48] S. H. Wang , P. Yang , X. F. Sun , H. L. Xing , J. Hu , P. Chen , Z. T. Cui , W. K. Zhu , Z. J. Ma , Appl. Catal. B‐Eviron. 2021, 297, 120386.

[49] S. H. Wang , P. Yang , X. F. Sun , H. L. Xing , J. Hu , P. Chen , Z. T. Cui , W. K. Zhu , Z. J. Ma , Adv. Energy Mater. 2020, 10, 1903854.

[50] G. H. Choi , N. C. S. Selvam , H. Kim , Y. S. Park , J. Jung , M. G. Nam , H. S. Jeon , Appl. Catal. B‐Eviron. 2023, 333, 122816.

[51] Y. H. Gan , M. L. Cui , X. P. Dai , Y. Ye , F. Nie , Z. T. Ren , X. L. Yin , B. Q. Wu , Y. H. Cao , R. Cai , X. Zhang , Nano Res. 2022, 1, 6.

[52] H. Liu , W. Lei , Z. Tong , K. Guan , Q. Jia , S. Zhang , H. Zhang , ACS Appl. Mater. Interfaces 2021, 13, 24604. 34027659 10.1021/acsami.1c01222

[53] F. Yu , H. Q. Zhou , Y. F. Huang , J. Y. Sun , F. Qin , J. M. Bao , W. A. Goddard , S. Chen , Z. F. Ren , Nat. Commun. 2018, 9, 2551. 29959325 10.1038/s41467-018-04746-z PMC6026163

[54] Q. Shi , Q. Liu , Y. Ma , Z. Fang , Z. Liang , G. Shao , B. Tang , W. Y. Yang , L. Qin , X. S. Fang , Adv. Energy Mater. 2020, 10, 1903854.

[55] Y. Pan , K. Sun , S. Liu , X. Cao , K. Wu , W. Cheong , Z. Chen , Y. Wang , Y. Li , Y. Liu , D. Wang , Q. Peng , C. Chen , Y. Li , J. Am. Chem. Soc. 2018, 140, 2610. 29341596 10.1021/jacs.7b12420

[56] D. Li , Z. Y. Y. Li , R. Zou , G. Shi , Y. M. Huang , W. Yang , W. Yang , C. F. Liu , X. W. Peng , Appl. Catal. B‐Eviron. 2022, 307, 121170.

[57] L. Li , P. Wang , Q. Shao , X. Huang , Chem. Soc. Rev. 2020, 49, 3072. 32309830 10.1039/d0cs00013b

