# Supplementary material for: Hollow Spherical Heterostructured FeCo‐P Catalysts Derived from MOF‐74 for Efficient Overall Water Splitting
Source: Adv Sci (Weinh). 2023 Nov 20;11(2):2306919. doi: 10.1002/advs.202306919 (PMC10787075; doi:10.1002/advs.202306919)
Supplement: Supplementary file 1 — Supporting information [file ADVS-11-2306919-s001.pdf]

ADVANCED SCIENCE
Open Access

Supporting Information

for *Adv. Sci.*, DOI 10.1002/advs.202306919

Hollow Spherical Heterostructured FeCo-P Catalysts Derived from MOF-74 for Efficient Overall Water Splitting

Hualin Jiang, Zhe Zhao, Gang Li, Mengxue Wang, Pinghua Chen, Xiaotian Liu, Xinman Tu, Yitian Hu, Zhen Shen and Yirou Wu*

Supporting information

1. Characterizations

The morphology and structure of the material were characterized using scanning electron microscopy (SEM, ZEISS Gemini 300) and transmission electron microscopy (TEM, Tecnai G2F30) operated at 200 kV, including high-resolution transmission electron microscopy (HRTEM), selected area electron diffraction (SAED) imaging, and energy-dispersive X-ray spectroscopy (EDS) elemental mapping analysis. X-ray photoelectron spectroscopy (XPS) data were collected using the Kratos XSAM-800 spectrometer to analyze the elemental composition and surface oxidation states. The crystalline phase structure of the material was investigated using an X-ray diffractometer (XRD, Bruker AXS D8) equipped with a Cu-Kα radiation source. The specific surface area and pore structure of the samples were analyzed using the Brunauer-Emmett-Teller (BET) analyzer (Tristar II 3020) from the United States.

2. Electrochemical measurements.

Electrochemical performance testing was conducted using the Shanghai Chenhua Electrochemical Workstation (CHI660E). Prior to OER or HER testing, oxygen or nitrogen gas was purged into a 1 mol L^{-1} KOH solution for at least 10 minutes to ensure saturation of the solution with oxygen or nitrogen. A standard three-electrode system was employed: a catalyst (1×1 cm^2) prepared on a NF substrate served as the working electrode, graphite served as the counter electrode, and Ag/AgCl was used as the reference electrode. The electrolyte used was a 1.0 mol L^{-1} KOH solution (pH=14).

The preparation process for the Pt/C and IrO_2 working electrodes, used as the anode and cathode materials in the control experiments, is as follows: The NF (1×2 cm^2) was sequentially ultrasonically cleaned in acetone, dilute hydrochloric acid, anhydrous ethanol, and deionized water to remove surface oxides. After vacuum drying, it was used as the catalyst support. 5 mg of Pt or IrO_2 was dispersed in a mixture consisting of 500 μL of ethanol, 500 μL of deionized water, and 20 μL of Nafion solution (5 wt%). Ultrasonication was used to create a homogeneous solution. 100 μL of this uniform solution was evenly coated onto 1×1 cm^2 NF. After drying, the working electrodes were obtained.

When performing LSV polarization curve tests, the scan rate was maintained at 5 mV s^{-1}, and iR compensation was applied. The applied potential was calibrated against the reversible hydrogen electrode (RHE).

$$E_{RHE} = E_{Ag/AgCl} + 0.059\ pH + E^{\theta}_{Ag/AgCl} \quad (S1)$$

$E^{\theta}_{Ag/AgCl}$ represents the electrode potential of the reference electrode, with $E^{\theta}_{Ag/AgCl}$ = 0.197 V, and subsequently, calculations were performed based on the following formula.

$$\eta = E_{vs.RHE} - E_{Theory} \quad (S2)$$

The overpotential was calculated, where the theoretical potentials for OER and HER relative to the RHE were 1.23 V and 0 V, respectively. The Tafel slope was obtained using the following equation:

$$\eta = a + b \log j \quad (S3)$$

Where η represents the overpotential, b is the Tafel slope, and j represents the current density.

Electrochemical active surface area (ECSA) : The ECSA was calculated using the C_{dl} value, which was determined from the CV curves obtained through conversion calculations. The non-Faradaic region within the voltage range was chosen, with a voltage window of -0.29 to -0.23 V. CV experiments were conducted at different scan rates (100, 150, 200, 250, and 300 mV s^{-1}) within the range of -0.29 to -0.23 V. By plotting the difference in current density (the difference between anodic and cathodic current densities) against the scan rate and fitting the resulting curve, the slope represents the double-layer capacitance (C_{dl}), calculated as follows: $C_{dl} = I/\nu$, where I is the current density in mA cm^{-2}, and ν is the scan rate in mV s^{-1}.

The electrochemical impedance spectroscopy (EIS) tests were conducted using a three-electrode system on the CHI 660E electrochemical workstation, with a testing frequency range from 0.1 to 10^5 Hz. The working electrode for impedance measurements was prepared as follows: Initially, indium tin oxide (ITO) conductive glass (1 cm×3 cm) was pretreated by ultrasonication in acetone and deionized water for 1 hour each, followed by a final ultrasonication step in anhydrous ethanol for 2 hours to remove impurities from the surface of the conductive glass. After drying in an oven, 5 mg of the material was dispersed in a mixture of 300 μL anhydrous ethanol/water/0.05% Nafion (V : V : V = 1 : 1 : 1). A 50 μL sample solution was evenly loaded onto the conductive glass (1 cm×1 cm) and allowed to dry naturally. The electrolyte solution used for testing consisted of 2.5 mmol L^{-1} potassium ferrocyanide ($K_4Fe(CN)_6 \cdot 3H_2O$), 2.5 mmol L^{-1} potassium ferricyanide ($K_3[Fe(CN)_6]$), and 0.5 mol L^{-1} sodium sulfate (Na_2SO_4) solution.

Electrochemical stability testing was conducted using two methods. The first involved CV with multiple cycles, followed by testing the LSV curves initial and after the scans. The second method utilized chronoamperometry (i-t), where a fixed current density under a certain voltage was maintained for testing, typically selecting stability at a specific current density

(e.g., 10 mA cm^{-2}) over a 24-hour duration. The durability of the catalyst was assessed by measuring changes in current density initial and after 24 hours of testing, with smaller changes indicating greater catalyst stability.

3. Density functional theory (DFT) calculation

DFT calculations were performed using the Vienna ab-initio simulation package (VASP) [1] to study the electrolysis process of H_2O. We employed a kinetic energy cutoff of 400 eV and projector-augmented waves [2] and conducted DFT calculations using the Perdew-Burke-Ernzerhof (PBE) functional [3]. Corrections for van der Waals interactions were applied using the DFT-D3 method [4]. The FeP(110) and Co_2P(130) surfaces were cleaved with a vacuum layer of 15 Å to create the surface structures of FeP and Co_2P, respectively. In the case of the Co_2P/FeP heterojunction structure, only one layer of Co_2P(130) was placed on the FeP(110) surface. The bottom two layers of all models were fixed to simulate the bulk phase. All models underwent full relaxation with an energy convergence criterion of 10^{-5} eV and a force convergence criterion of 0.02 eV $Å^{-1}$. The Monkhorst-Pack k-point grid with a resolution of 0.04 $Å^{-1}$ was used for Brillouin zone integration. The formula for calculating the adsorption energy (E_{ads}) is as follows:

$$E_{ads} = E_{total} - E_{substrate} - E_{adsorbate} \quad \text{(S4)}$$

Where E_{total}, $E_{substrate}$ and $E_{absorbate}$ represent the energies of the adsorption structure, substrate, and adsorbate, respectively. The formula for calculating the Gibbs free energy is as follows:

$$G = E_{DFT} + ZPE - TS \quad \text{(S5)}$$

Where G, E_{DFT}, ZPE, and TS represent the Gibbs free energy, free energy obtained from DFT calculations, zero-point energy, and entropy contribution, respectively.

4. Figures S1-S9.

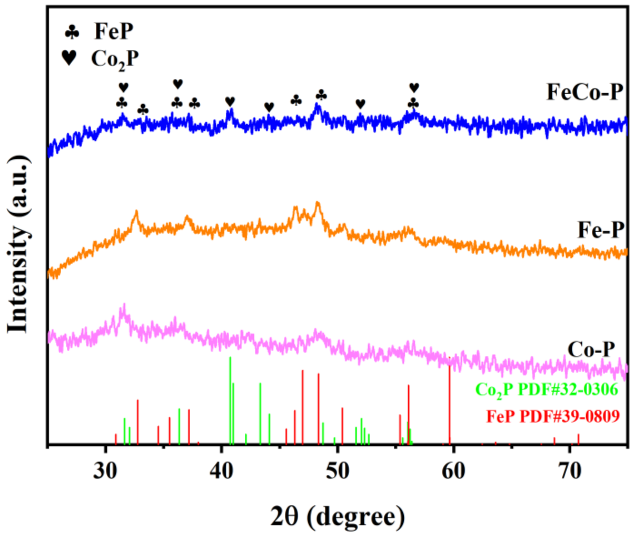

Figure S1. XRD spectrum of Co-P、Fe-P and FeCo-P

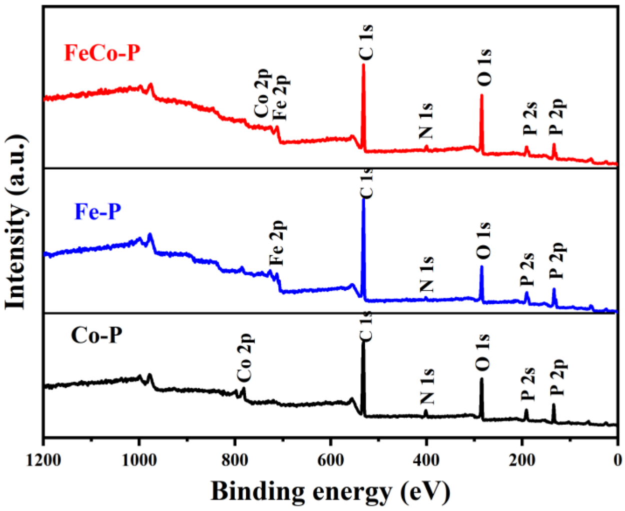

Figure S2. XPS survey spectrum of Co-P、Fe-P and FeCo-P.

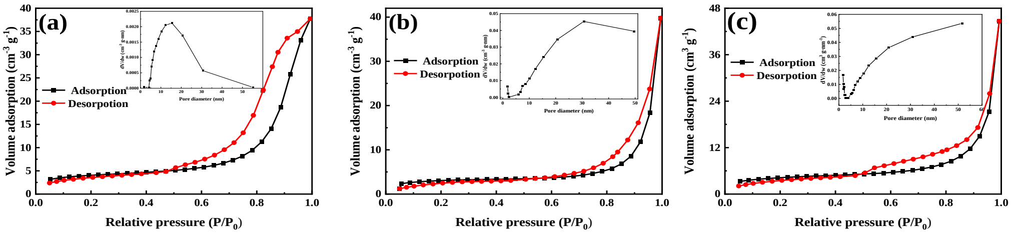

Figure S3. Nitrogen adsorption-desorption isotherms and pore size distribution of (a) Fe P, (b) CoP, (c)FeCo-P.

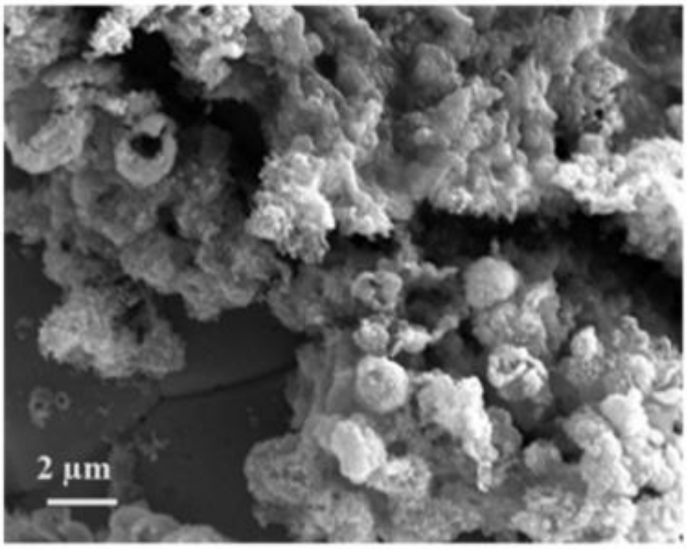

Figure S4.SEM of FeCo-P after 24 h HER durability test.

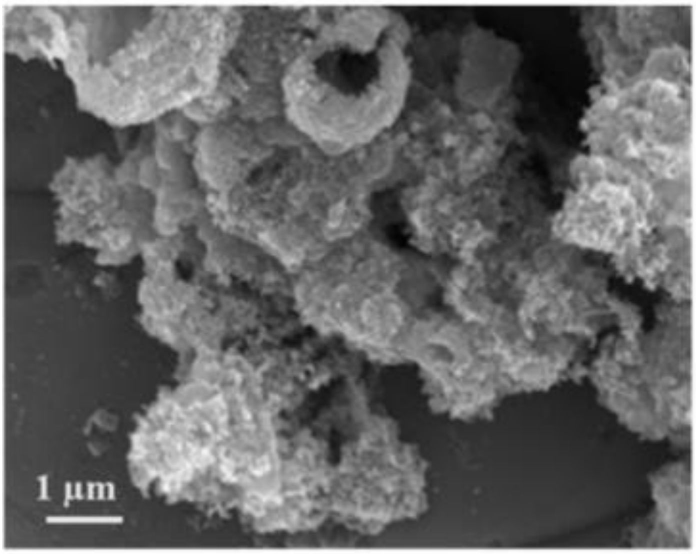

Figure S5.SEM of FeCo-P after 24 h OER durability test.

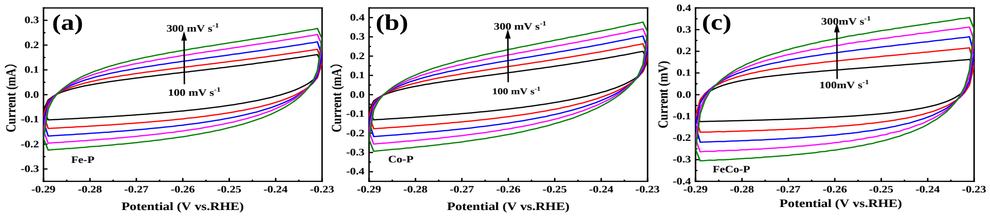

Figure S6. CV curves at different scanning rates of (a) FeP, (b) CoP, (c)FeCo-P.

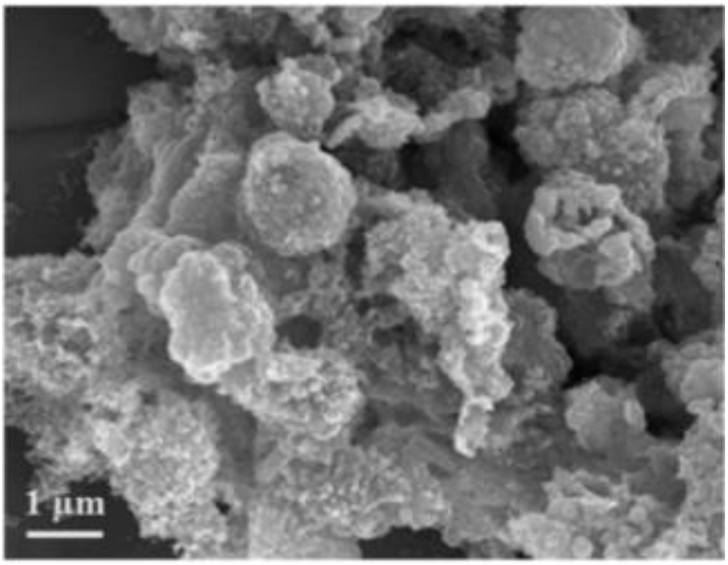

Figure S7. SEM of FeCo-P after 24 h OWS durability test.

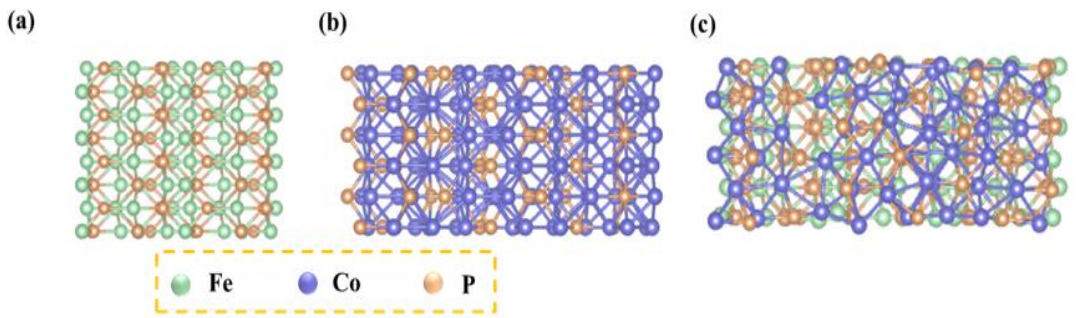

Figure S8.(a) Simulated surface structure of FeP(Fe-P). (b) Simulated surface structure of Co_2P(Co-P). (c) Simulated surface structure of FeP/Co_2P(FeCo-P);

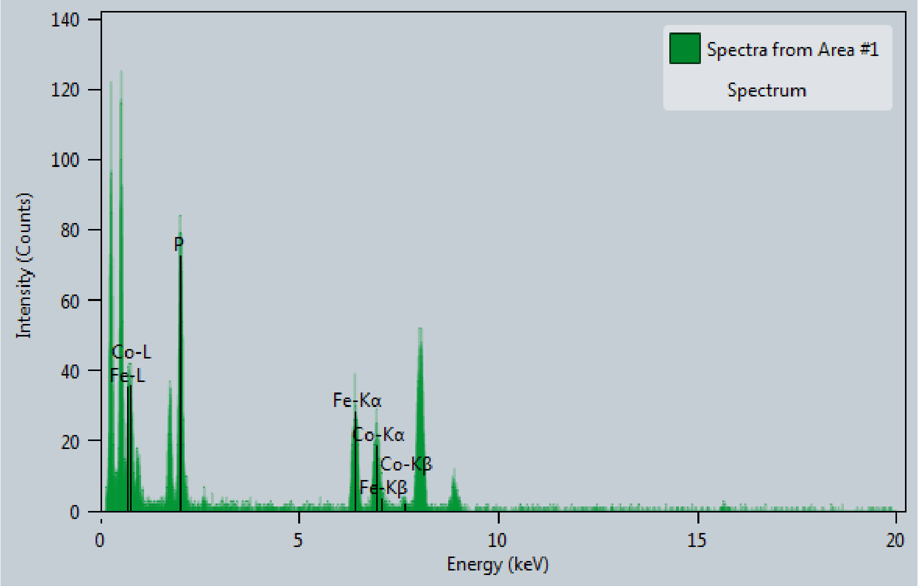

Figure S9. Energy dispersion spectrometer of FeCo-P

5. Tables S1-S5.

Table S1. BET surface area, pore volume and pore size of Fe-P, Co-P and FeCo-P.

	Fe-P	Co-P	FeCo-P
Surface area (m^2 g^{-1})	10.2	13.6	14.3
Average pore diameter (nm)	13.6	17.1	21.5

Table S2. TOF values (HER) for Fe-P, Co-P and FeCo-P.

Samples	TOF (H_2 s^{-1} per Co atom)	TOF (H_2 s^{-1} per Fe atom)	TOF (H_2 s^{-1} per M atom)
Fe-P		0.012	0.012

Co-P	0.026		0.026
FeCo-P	0.109	0.025	0.134

Table S3. Table of Comparison between FeCo-P and reported electrocatalysts for HER in 1.0 mol L^{-1} KOH.

Materials	Overpotential @ j=10 mA cm^{-2} (mV)	Tafel slope (mV dec^{-1})	References
FeP/Ni_2P	150	24.2	[5]
CoP@CoOOH	81.7	50.7	[6]
Ru-NiFe-P	44	80	[7]
FeCo/Co_2P@NPCE	260	120	[8]
Ni-Mo-P	69	108.4	[9]
CoFeP NS@NCNF	113	108	[10]
N−CoS_2/G	109	68.9	[11]
NiO/CoFe alloy	218	102.9	[12]
**FeCo-P**	**131**	**89.90**	**This work**

Table S4. Table of Comparison between FeCo-P and reported electrocatalysts for OER in 1.0 mol L^{-1} KOH.

Materials	Overpotential @ j=10 mA cm^{-2} (mV)	Tafel slope (mV dec^{-1})	References
FeP/Ni_2P	154	22.7	[5]
CoP@CoOOH	200	131.8	[6]
FeCo/Co_2P@NPCE	330	61	[8]
Ru, Ni-CoP	251	102.3	[13]
V-CoP	267	39	[14]
Fe-Co_2P@Fe-N-C	300	79	[15]
E-Mo-NiCoP	269	76.7	[16]
Co_xP/NC-Mel	312	52.2	[17]
NiCoP/CC	242	64.2	[18]

FeCo-P	240	38.24	This work

Table S5. Table of Comparison between FeCo-P and reported electrocatalysts for overall water splitting in 1.0 mol L^{-1} KOH at the current density of 10 mA cm^{-2}.

Materials	Cell Voltage (V)	References
$CoMoRu_{0.25}O_x$/NF	1.51	[19]
CoP/CoOOH	1.52	[5]
Ni-Mo-P	1.46	[9]
Ru-NiFe-P	1.47	[7]
CoFe PBA@CoP	1.542	[20]
Fe-Ni_5P_4/NiFeOH	1.55	[21]
Mo- CoFeLDH/NF	1.55	[22]
MZU-$Co_{2.5}Zr_1$	1.56	[23]
SnS/$NiCo_2O_4$	1.57	[24]
CoFeP NS@NCNF	1.59	[10]
V-CoP	1.59	[14]
CoFeO@N/S-rGO	1.63	[25]
FeCo/Co_2P@NPCE	1.68	[8]
**FeCo-P**	**1.49**	**This work**

References

[1] K. G, J.Furthmüller, *Phys. Rev. B.* **1996**, 54, 11169.

[2] G. Kresse , D. Joubert, *Phys. Rev. B.* **1999**, 59, 1758.

[3] J. P. Perdew, K. Burke, M. Ernzerhof, *Phys. Rev. Lett.* **1996**, 77, 3865.

[4] S. Grimme, J. Antony, S. Ehrlich, H. Krieg, *J. Chem. Phys.* **2010**, 132, 154104.

[5] F. Yu, H.Q. Zhou, Y. F. Huang, J. J. Sun, F. Qin, J. J. Bao, W. A. Goddard, S. Chen, Z.F. Ren, *Nat. Commun.* **2018**, 9, 2551.

[6] B. Zhang, J. W. Shan, W. L. Wang, P. Tsiakaras, Y. Y. Li, *Small.* **2022**, 18, 2106012.

[7] M. J. Qu, Y. M. Jiang, M. Yang, S. Liu, Q. F. Guo, W. Shen, M. Li, R. X. He, *Appl. Catal. B-Eviron.* **2020**, 263, 118324.

[8] Q. Shi, Q. Liu, Y. Ma, Z. Fang, Z. Liang, G. Shao, B. Tang, W. Y. Yang, L. Qin, X. S. Fang, *Adv. Energy Mater.* **2020**, 10, 1903854.

[9] B. Zhang, F. Yang, X. D. Liu, N. W, S. Che, Y. F. Li, *Appl. Catal. B-Eviron.* **2021**, 298, 120494.

[10] B. Wei, G. C. Xu, J. C. Hei, L. Zhang, T. T. Huang, Q. Wang, *J. Colloid Interface Sci.* **2021**, 602, 619.
[11] Y. Tong, Q. Sun, P. Z. Chen, L. Chen, Z. F. Fei, P. J. Dyson, *ChemSusChem.* **2020**, 13, 5112.
[12] Y. T. Lei, L. L. Zhang, D. N. Zhou, C. L. Xiong, Y. F. Zhao, W. X. Chen, X. Xiang, H. S. Shang, B. Zhang, *Renew. Energ.* **2022**, 194, 459.
[13] Y. Y. Song, J. L. Cheng, J. Liu, Q. Ye, X. Gao, J. J. Lu, Y. L. Cheng, *Appl. Catal. B-Eviron.* **2021**, 298, 120488.
[14] R. Zhang, Z. H. Wei, G. Y. Ye, G. J. Chen, J. J. Miao, X. H. Zhou, X. W. Zhu, X. Q. Cao, X. N. Sun, *Adv. Energy Mater.* **2021**, 11, 2101758.
[15] X. W. Lv, W. S. Xu, W. W. Tian, H. Y. Wang, Z. Y. Yuan, *Small.* **2021**, 17, 2101856.
[16] J. H. Lin, Y. T. Yan, C. Li, X. Q. Si, H. H. Wang, J. L. Qi, J. Cao, Z. X. Zhong, W. D. Fei , J. C. Feng, *Nanomicro Lett.* **2019**, 11, 1.
[17] H. T. Liu, S. X. Yang, J. Y. Ma, M. L. Dou, *Nano Energy.* **2022**, 98, 107315.
[18] C. Du, L. Yang, F. L. Yang, G. Z. Cheng, W. Luo, *ACS Catalysis.* **2017**, 7, 4131.
[19] D.Thiyagarajan, A. Thirumurugan, B. K. Lee, *Int. J. Hydrog. Energy.* **2022**, 47, 39908.
[20] L. Quan, S. H. Li, Z. P. Zhao, J. Q. Liu, Y. Ran, J. Y. Cui, W. Lin, X. L. Yu, L. Wang, Y. H. Zhang, J. H. Ye, *Small Methods.* **2021**, 5, 2100125.
[21] C. F. Li, J. W. Zhao, L. J. Xie, J. Q. Wu, G. R. Li, *Appl. Catal. B-Eviron.* **2021**, 291, 119987.
[22] G. Y. Zhao, B. W. Wang, Q. Yan, X. H. Xia, *J. Alloys Compd.* **2022**, 902, 163738.
[23] P. H. Chen, M. X. Wang, G. F. Li, H. L. Jiang, A. Rezaeifard, M. Jafarpour, G. H. Wu, B. Y. Rao, *Inorg. Chem.* **2022**, 61. 18424.
[24] G. John, S. Gopalakrishnan, A. Sharan, M. Navaneethan, J. Kulandaivel, N. Singh, P. J. Jesuraj, *Energy & Fuels.* **2022**, 37, 624.
[25] B. H. Zhang, H. X. Wang, Z. Zuo, H. S. Wang, J. T. Zhang, *J. Mater. Chem. A.* **2018**, 6, 15728.
